# The Influence of Prefabricated Foot Orthosis Use on the Modification of Foot Posture in Adults with Pronated Feet: A Randomised Controlled Trial

**DOI:** 10.3390/healthcare13020163

**Published:** 2025-01-16

**Authors:** María Victoria Cáceres-Madrid, Julián Fernando Calderón-García, Francisco José Rodríguez-Velasco, Belinda Basilio-Fernández, Fidel López-Espuela, Esperanza Santano-Mogena, Marina Fontán-Jiménez, Sergio Rico-Martín

**Affiliations:** 1 Department of Nursing, Podiatry, Centro Universitario de Plasencia, University of Extremadura, 10600 Plasencia, Spain; pgvicky@unex.es (M.V.C.-M.); bbasfer@unex.es (B.B.-F.); marinaf@unex.es (M.F.-J.); 2 Department of Nursing, Nursing and Occupational Therapy College, University of Extremadura, 10003 Cáceres, Spain; fidellopez@unex.es (F.L.-E.); esantano@unex.es (E.S.-M.); sergiorico@unex.es (S.R.-M.); 3 Department of Nursing, Faculty of Medicine and Health Science, University of Extremadura, 06005 Badajoz, Spain; fcorodriguezv@unex.es

**Keywords:** pronated foot, foot posture index, pronation, foot orthoses

## Abstract

**Background:** The use of foot orthoses to treat different pathologies in pronated feet in adults is widespread among podiatric professionals, although it has not been conclusively demonstrated to modify foot posture in the short or medium term. **Objective:** The aim of this study was to evaluate whether prefabricated foot supports reduce pronated foot posture in adults, as measured by the foot posture index (FPI). **Methods:** A randomised controlled clinical trial was conducted in 109 subjects with pronated feet. The participants were randomly placed into a control group that did not receive any intervention and an experimental group that used prefabricated orthoses for 6 months. The changes in the FPI were evaluated in both groups at 6 months. **Results:** Over the six-month follow-up period, the delta FPI variable was changed by −1.1 ± 2.2 points in the experimental group, whereas the same variable was reduced by 1.2 ± 2.1 points in the control group (*p* = 0.001). The participants in the experimental group neutralised their FPIs significantly more than those in the control group did (39.3% vs. 8.5%; *p* = 0.041). Moreover, individuals in the experimental group were more likely to migrate from highly pronated feet to pronated feet than those in the control group were (45.8% vs. 20%; *p* < 0.001). Finally, multivariate analysis indicated that prefabricated foot orthoses were associated with an improved FPI (OR: 6.23, CI%95: 2.72–17.09; *p* < 0.001). However, the corrective effect provided by the prefabricated foot orthoses, which neutralised the pronated posture, was nullified by the presence of index minus metatarsal formula. **Conclusions:** The use of prefabricated orthoses resulted in a decreased FPI in adults, especially in those with highly pronated feet. However, the index minus presence nullified the effect of prefabricated orthoses on foot posture neutralisation.

## 1. Introduction

Pronation is a physiological movement of the foot that is essential in human gait [1]. The pronated position, characterised by calcaneal eversion and plantar flexion along with the adduction of the talus, results in the flattening of the medial longitudinal arch [2]. This hyperpronation and flattening of the medial longitudinal arch can lead to deformities in the first metatarsal–phalangeal segment [3,4] or central metatarsalgia [5]. Certain morphological factors, such as the index minus metatarsal formula, which is more prevalent in individuals with pronated feet, are associated with the development of pronated feet [6,7,8,9]. Treatment varies depending on the severity and reducibility of the condition, but the most widely accepted treatment by podiatry professionals is foot orthoses [10,11]. Custom foot orthoses require specific moulding techniques and different equipment for their fabrication. However, prefabricated orthoses, created from a standard mould, have a general shape, reduce the time required for treatment fabrication, and have demonstrated similar effectiveness in reducing hindfoot eversion [12]. Although the clinical mechanism determining the effectiveness of prefabricated orthoses in the adult population with pronated feet has not been fully elucidated, their use is widespread [13]. However, the medium-term effect of orthotic treatment implementation in asymptomatic adults with pronated feet, when combined with foot muscle exercises or within shorter periods, remains unknown [14]. Short-term studies have shown reduced pressures on the medial longitudinal arch and decreased hyperpronation [15]. Considering that, in an adult population with pre-existing pronated feet, the progression of this pronation is influenced by the reduced functional capacity of foot joint biomechanics [16].

Therefore, the aim of this randomised clinical trial was to assess foot posture index (FPI) changes in patients with pronated and highly pronated feet after six months of the use of prefabricated plantar orthoses and to analyse the predisposing factors.

## 2. Methods

### 2.1. Study Design

A double-blind randomised controlled clinical trial (RCT) was conducted according to the CONSORT 2010 guidelines [17]. The study protocol was previously registered on clinicaltrials.gov (NCT03954821). The Bioethics Committee of the University of Extremadura (ID: 07/2017) provided a positive review of the research project, approving the study procedures. All participants provided written informed consent, and the study adhered to the principles outlined in the Declaration of Helsinki. Patient confidentiality was maintained at all times.

#### 2.1.1. Setting

This study was carried out at the facilities of the podiatric clinic of the University of Extemadura (Plasencia, Spain) between October 2016 and June 2017.

#### 2.1.2. Participants

Individuals over 18 years of age with pronated and/or highly pronated feet were selected according to the criteria established by the FPI described and validated by Redmond et al. [18]. Participants were assessed.

The inclusion criteria for the study were (a) having pronated feet in the static position measured by FPI, (b) having asymptomatic feet (no reported pain in the midfoot in static or dynamic positions), (c) absence of joint deformities that could hinder accurate foot measurements (structural asymmetries of the foot and leg, amputations), (d) aged between 18 and 50 years, and (e) providing signed informed consent.

The exclusion criteria were (a) suffering from degenerative osteoarticular deformities, (b) having undergone surgical interventions on the lower extremities, specifically on the foot (Hallux Abductus Valgus surgeries, metatarsal surgeries, fixations due to foot and ankle fractures that impede normal mobility), (c) experiencing balance loss that hinders accurate foot measurement, (d) having painful keratopathies or plantar warts that hinder foot support, (e) current use of other orthopaedic treatments (foot orthoses), (f) inability to maintain a bipedal position for an extended period, and (g) inability to maintain a coordinated posture on the examination bench to assess foot posture.

No previous similar studies have been published; therefore, the sample size was calculated anticipating a Cohen’s δ = 0.40 (medium effect size) with β = 0.80 and α = 0.05 to detect changes in the FPI at 6 months. The minimum sample size required was 52 subjects per group.

#### 2.1.3. Randomisation and Blinding

The participants were randomly assigned to either the control group (which would not receive any intervention) or the experimental group, which would use a pair of prefabricated orthoses. Randomisation was performed via Epidat 3.1 software (Epidat: programa para análisis epidemiológico de datos. Consellería de Sanidade, Xunta de Galicia, España). The randomisation sequence remained hidden until the intervention. After enrolment and baseline data collection, the investigator in charge of randomisation contacted the podiatrist in charge of the intervention. The principal investigator and the podiatrist who performed the measures did not know the assigned group of participants.

#### 2.1.4. Intervention

**Figure 1:**
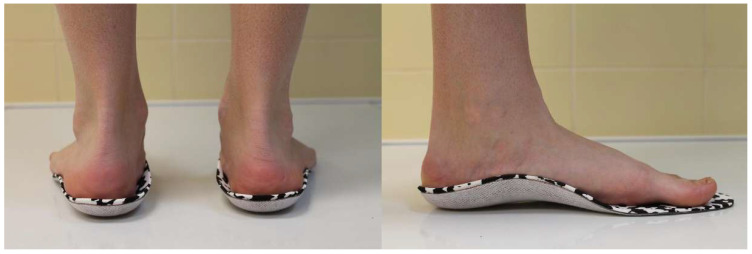
Prefabricated orthoses used this study.

The experimental group received a treatment consisting of a pair of prefabricated orthoses (unmodified foot orthoses) made from resins and ethylene vinyl acetate (E.V.A.) materials, with a white and black inner lining that comes into contact with the foot (Herbitas^®^, Valencia, Spain) (Figure 1). These insoles are designed with an internal longitudinal arch that aligns with the individual’s foot arch corresponding to the size of each participant’s foot.

Each participant was initially provided with the insoles, ensuring the correct adaptation of the treatment. Upon signing the informed consent form, they were asked to commit to using suitable footwear along with foot orthoses for at least 7 h a day for a minimum of five days per week. The control group did not undergo any intervention and remained subject to a subsequent evaluation of their foot posture.

A follow-up was conducted via email every month to ensure proper utilisation of the treatment or to address any potential issues arising from it over a period of 6 months.

Only one podiatrist conducted the intervention; no other researchers knew the assigned group of the participants.

#### 2.1.5. Measurements

A podiatrist with more than 10 years of experience in the treatment of foot pathologies oversaw all the measurements. All measurements were performed early in the morning with participants standing with equally distributed weight on both feet, dressed in shorts to facilitate the examination and barefoot. All participants indicated that they had not been physically active in the 8 h prior to the examination.

### 2.2. Primary Outcome

During the FPI test, the subject must remain standing upright with relaxed foot posture. The following six criteria were used [18]: talar head palpation, supra and infra lateral malleolar curvature, eversion and inversion of the calcaneus, prominence in the region of the talonavicular joint, congruence of the medial longitudinal arch, and abduction and adduction of the forefoot on the rear foot. Each item is given a score between −2 and +2, adding up to a total score of −12 to +12. The foot can be categorised into the following groups: highly pronated (+10 to +12), pronated (+6 to +9), normal (0 to +5), supinated (−1 to −4), and highly supinated (−5 to −12).

### 2.3. Study Variables

Age, sex, and shoe size were self-reported by the subjects. Weight (in kilograms) and height (in centimetres) were measured via a highly precise mechanical column scale with sliding weights combined with a telescopic stadiometer. Body mass index (BMI) was calculated by dividing weight by the square of height (kg/m^2^).

Metatarsal length was assessed via the Spooner method [19], which measures the relative length of the metatarsals from the navicular tuberosity to the metatarsal heads via a caliper [20]. Additionally, foot types were classified as Egyptian, Greek, or square based on observations of the digital formula [21].

At the end of the 6-month period, the foot posture was reassessed following the previously described protocol for both the control and experimental groups.

### 2.4. Statistical Analysis

All values are expressed as the means ± standard deviations, frequencies, and percentages. A normal distribution was confirmed by the Kolmogorov-Smirnov test, and homogeneity was confirmed by Levene’s test before the standard tests were applied. For quantitative variables, comparative analysis was performed for each group via paired *t* tests (comparisons within the same group at baseline and at six months) and independent *t* tests (comparisons between the control and experimental groups). When there was no normality, the Wilcoxon test and the Mann-Whitney U test were used. To preserve data independence [22], and considering a strong correlation between the FPI scores of the left and right feet in healthy individuals, data from only one foot (the randomly chosen left foot) were included in the statistical analyses, despite measurements being taken for both feet. Categorical variables were compared with Pearson’s chi-square test or Fisher’s test, as well as McNemar’s test (to compare the pronated or highly pronated migrations within the same group). Additionally, univariate and multivariate logistic regression analyses were conducted to explore the possible relationships between the dependent variables (migration from highly prone foot to pronated foot or from pronated foot to neutral foot) and the independent variables. Odds ratios (ORs) and 95% confidence intervals (CIs) were estimated. The multivariate analysis included the independent variables, for which *p* < 0.10 was obtained in the univariate analysis.

All analyses were conducted via IBM SPSS Statistics version 24 software, and the significance threshold considered was *p* < 0.05.

## 3. Results

**Figure 2:**
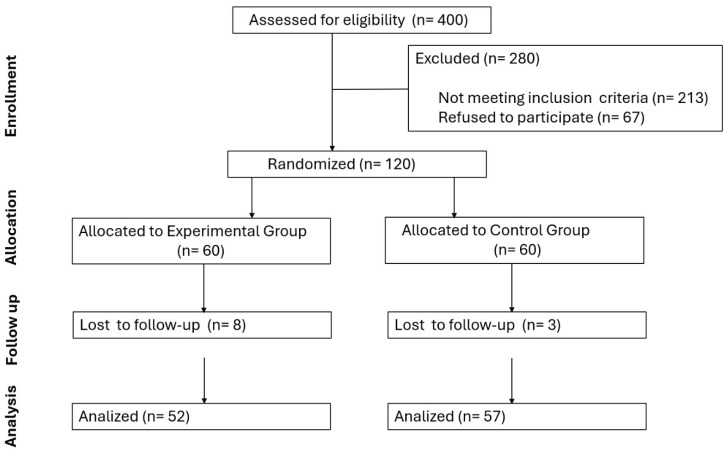
Flow chart of study patients.

A total of 400 patients were initially evaluated. A total of 120 patients with pronated or highly pronated feet who met the inclusion criteria and signed the consent form were randomised (60 per group). However, 11 subjects (control group = 3 and intervention group = 8) were included. Finally, 109 patients completed the study. A detailed flow chart is presented in Figure 2.

At the beginning of the study, among all participants (*n* = 109), 82.4% in the control group exhibited a pronated foot posture, whereas 17.5% had a highly pronated foot posture. Within the experimental group, 53.8% had a pronated foot, and 46.1% had a highly pronated foot. There was a significant difference (*p* = 0.001) in the distribution of groups, with a notably greater number of highly pronated feet in the experimental group (Table 1). No statistical differences were observed in the rest of the descriptive variables analysed according to the assigned group.

At the 6-month follow-up, subjects in the experimental group experienced a change in the delta FPI (FPI post/pre) variable of −1.1 ± 2.2 points, whereas subjects in the control group experienced an increase of 1.2 ± 2.1 points in the same variable, and this difference was statistically significant (*p* = 0.001). Compared with those in the control group, the FPIs for each criterion were significantly lower in the experimental group for the supra and infra lateral malleolar curvature criteria (*p* = 0.032), the calcaneus bisecting in the frontal plane (*p* = 0.002), the prominence of the talonavicular region (*p* = 0.004), the congruence of the medial longitudinal arch (*p* = 0.001), and forefoot abduction/adduction with respect to the hindfoot (*p* < 0.001). However, the criterion for the palpation of the head of the talus did not differ between the groups (Table 2).

**Figure 3:**
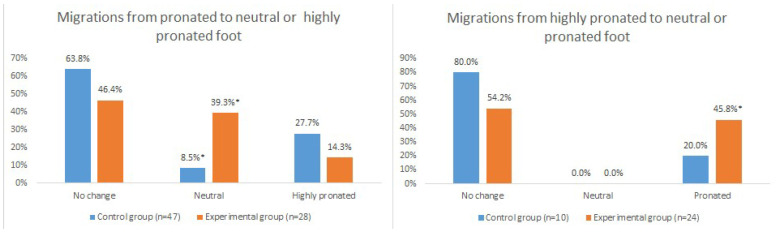
Control and experimental group participant migrations at the end of the study. * *p* < 0.05.

The FPIs of only 4 (8.5%) participants in the control group with a pronated posture were neutralised, whereas, in the experimental group, 11 (39.3%) participants transitioned from a pronated posture to a neutral posture. Another 11 (45.8%) participants witnessed a reduction in their foot posture, shifting from the initially highly pronated posture to a pronated posture (Figure 3)

In Table 3, the factors influencing foot posture changes are presented. Upon conducting univariable and multivariable analyses to identify potential predictors for the shift from highly pronated to pronated or neutral foot posture, it was observed that only the use of foot orthoses (*p* < 0.001) was significantly associated with this change in foot posture.

To assess the influence of the use of prefabricated foot orthoses and other predisposing factors for the shift from pronated to neutral foot posture, Table 4 indicates that, in the univariable analysis, both the use of foot orthoses and the metatarsal index minus formula were associated with this change. However, in the multivariable analysis, this influence disappeared. The corrective effect provided by the use of prefabricated foot orthoses to neutralise the pronated posture in the subjects of the experimental group was nullified in these cases by the presence of the index minus.

Finally, an additional analysis was performed to find the possible association of the FPI changes according to the metatarsal formula status in both groups (Table 5). We observed that participants with index plus and index minus plus experienced higher variations in the FPI item scores and in the final FPI score than participants with a higher FPI score.

## 4. Discussion

The main findings of this study indicated that the use of the prefabricated foot orthoses significantly reduced the FPI score and increased the migration rate of highly pronated to pronated feet and pronated feet to neutral feet.

Pronated and highly pronated feet are very common in the general population. Similar trends have been reported previously [23], indicating that, in a population exhibiting asymptomatic highly pronated feet, the prevalence of this posture is lower than that in a population with lower FPI scores under the same conditions. Similar results have been found in our study.

Previous studies in adult populations with highly pronated feet treated with custom foot orthoses have shown cases where the pronated posture did not improve [24,25]. The use of prefabricated insoles reduced both pronated and highly pronated foot postures. These changes were observed over a 6-month treatment period using prefabricated foot orthoses specifically designed for this study. However, Jafarnezhadgero et al. [26] also demonstrated improvements in supporting the medial longitudinal arch after 4 months of using foot orthoses. Prefabricated insoles have shown benefits such as reducing pressure on the hallux, toes, midfoot, and heel, similar to custom-made insoles, as shown by Lucas-Cuevas et al. [27] in a study involving long-distance athletes. In additional research, the easy use of prefabricated foot orthoses was found to decrease chronic lower back pain after 6 months of treatment [28]. In runners in which the effect of prefabricated orthoses on the prevention of running injuries was studied, it was only possible to establish that they improved the comfort of the runner [29], although we do not know whether the orthoses used in this study led to changes in pronation. Even though the participants in our trial were asymptomatic, the trend toward foot deformity tended to progress with age in the absence of any intervention, leading to a decrease in functional capacity within foot joint biomechanics [16].

There are discrepancies in the published results regarding the efficacy of foot orthoses in the treatment of flatfoot in adults [13], and the current literature lacks sufficient studies that demonstrate the effectiveness of foot orthoses in treating flat feet in adults. This lack of data is related to the absence of information regarding the type of foot orthoses used, the diagnostic process employed, and even the duration of treatment. Therefore, our study could represent progress in this area of research, as it demonstrates how a population diagnosed with pronated and highly pronated feet through the FPI can change their posture by using prefabricated insoles. Considering that the benefits obtained with custom foot orthoses do not appear to be significantly greater than those achieved with prefabricated insoles [30,31], prefabricated orthoses guarantee patient fit and proven treatment.

The number of subjects whose posture improved positively was influenced by the use of prefabricated insoles. However, the metatarsal index minus formula was the factor that negatively influenced individuals’ pronation. This suggests that prefabricated foot orthoses have a positive effect, which is supported by other studies [32] where the use of prefabricated insoles improved the posture and mobility of the first metatarsophalangeal joint.

Therefore, we can consider this metatarsal morphology as a predisposing factor for pronated feet, which is sufficiently influential in the unfavourable evolution of pronation. Thus, by analysing the results of our research, we can corroborate, as previously reported by the authors in [6,7,8,9], that the presence of the metatarsal index minus is the most prevalent metatarsal formula in the population with pronated feet due to the greater relative length of the first metatarsal compared to the other metatarsals, which affects the loading dynamics of the foot. In pronated feet, metatarsal index minus alters the biomechanics of the longitudinal and transverse arch, increasing the forces on the lateral metatarsals, which can exacerbate pathologies such as metatarsalgia [7]. This alteration could make it difficult for prefabricated orthoses to adequately redistribute loads, compromising treatment efficacy [8].

Several authors have concluded that FPI can be modified with specific therapeutic exercise programs [24,33]. Other authors who combined short-foot exercises with insoles reported significant modifications in the FPI score (MD: −0.67; 95% CI: −0.98 to −0.36; *p* < 0.0001) and recommended them as beneficial dynamic support when flatfoot problems are present [34]. This could justify the use of this type of treatment in combination with the orthoses used in our study for the treatment of different pathologies related to pronation, although there are no studies that address this aspect owing mainly to the difficulty that FPI does not correlate with dynamic barefoot examination [35]. Dynamic examinations are very suitable for quantifying the effects of pronation.

When prefabricated orthoses are prescribed, there is a reduction in the cost associated with their production, including materials and labour, as well as expenses related to patient examination, assessment, and follow-up visits. Additionally, prefabricated insoles do not require modifications but rather simple adjustments to the subject’s footwear.

The orthopaedic treatment of pronated feet is a controversial issue in children [36,37]. Some podiatrists consider asymptomatic pronated feet as a physiological variable and suggest treating it only if it has persistent symptomatology, as it is self-correcting and resolves spontaneously with age in most cases [38,39,40]. Other authors advocate preventive realignment in asymptomatic pronated feet, with no evidence that these feet become symptomatic without treatment [37,41,42]. In addition, some studies indicate that, in adolescence and adulthood, pronated feet may be associated with plantar fasciitis, plantar achilles, tibialis posterior tendonitis, hallux abductus valgus, hallux rigidus, chondromalacia patellae, and patellofemoral pain syndrome [37,43]. A recent systematic review has concluded that there is a lack of evidence on the effect of foot orthoses for flat or pronated feet in adults [13], showing the importance of randomised controlled trials such this study.

Although our study provides interesting data on the use of prefabricated orthotics in individuals with asymptomatic pronated feet, multicentre studies are needed to confirm our findings and provide more statistical power to ensure more accurate treatment.

### Limitations

One of the limitations of this study was the verification of usage by the experimental group, which ensured that the participants wore foot orthoses for a minimum of 7 h daily for at least 5 days a week, and they were evaluated alongside the control group. It would be necessary to ascertain whether increasing the duration of use of foot orthoses or extending the follow-up period would yield new outcomes, or if the degree of pronation evolution correlates with the duration of treatment usage. In addition, FPI is a static measurement and the foot is a dynamic structure, so our findings may vary if we use a dynamic method to detect pronation. On the other hand, the inter- and intra-rater reliability was not calculated because only a single measurement by a single operator was performed in each period. However, reliability has been analysed by several authors [18,44,45]. We considered the measurements to be correct because the podiatrist who carried them out had more than 10 years of experience in measuring FPI. Finally, we did not collect the physical activity status, so this variable was not considered in the analysis, which may influence our findings.

## 5. Conclusions

The use of prefabricated foot orthoses appears to be effective in reducing pronation in the short term. Thus, for patients with pronated feet, this type of orthosis could become the first-choice treatment, whereas customised orthoses could be reserved for symptomatic feet or when prefabricated orthoses do not demonstrate clinical efficacy. In patients with an index minus, owing to the limited observed effect in these participants to neutralise their posture, customised orthoses could be initially prescribed.

## Figures and Tables

**Table 1:** Baseline characteristics of the participants.

	Total Group *n* = 109	Control Group *n* = 57	Experimental Group *n* = 52	*p*-Value
Female (sex)	90 (82.6%)	47 (52.2%)	43 (47.7%)	0.432
Male (sex)	19 (17.4%)	10 (52.6%)	9 (47.3%)	0.675
Age (years)	24.5 ± 3.8	24.9 ± 3.5	23.9 ± 4.05	0.172
Weight (kg)	59.8 ± 11.2	59.3 ± 12.6	60.5 ± 9.2	0.523
Height (cm)	1.6 ± 0.8	1.3 ± 0.7	1.6 ± 0.8	0.723
BMI (kg/m^2^)	22.1 ± 3.01	21.95 ± 3.12	22.5 ± 2.85	0.332
Shoe size	38.6 ± 2.4	38.6 ± 2.5	38.6 ± 2.4	0.812
Metatarsal F.				
Index plus	44 (40.4%)	20 (35.1%)	24 (46.2%)	0.332
Index plus minus	15 (13.8%)	7 (13.2%)	8 (15.4%)	
Index minus	50 (45.9%)	30 (52.6%)	20 (38.5%)	
Digital F.				
Greek	78 (71.6%)	39 (69.4%)	39 (75.0%)	0.614
Egyptian	17 (15.6%)	9 (15.8%)	8 (15.4%)	
Square	14 (12.8%)	9 (15.8%)	5 (9.6%)	
FPI				
Pronated	75 (68.8%)	47 (82.4%)	28 (53.8%)	0.001
Highly pronated	34 (31.2%)	10 (17.5%)	24 (46.1%)	

**Table 2:** Control and experimental groups' FPI criteria 6 months later.

FPI Criteria	Group	∆ FPI Score ± SD	*p*-Value
Talar head palpation	Control	0.15 ± 0.49	0.329
	Experimental	0.05 ± 0.57	
Supra and infra lateral malleolar curvature	Control	0.21 ± 0.55	0.032
	Experimental	−0.01 ± 0.54	
Calcaneal frontal plane position	Control	0.22 ± 0.65	0.002
	Experimental	−0.21 ± 0.77	
Prominence in the region of the talonavicular joint	Control	0.15 ± 0.56	0.004
	Experimental	−0.15 ± 0.53	
Congruence of the medial longitudinal arch	Control	0.19 ± 0.54	0.001
	Experimental	−0.17 ± 0.58	
Abduction/adduction of the forefoot on the rearfoot	Control	0.22 ± 0.56	<0.001
	Experimental	−0.44 ± 0.63	
Total FPI score	Control	1.24 ± 2.14	0.001
	Experimental	−1.01 ± 2.26	

**Table 3:** Influential factors in the change in posture from highly pronated to pronated or pronated to neutral posture.

	Correct to Pronated or Neutral *n* = 28	No Change *n* = 81	Univariable Analysis		Multivariable Analysis	
			OR (IC 95%)	*p*-Value	OR (IC 95%)	*p*-Value
Foot orthoses	22 (78.6%)	30 (37.0%)	6.23 (2.72–17.09)	<0.001	6.23 (2.72–17.09)	<0.001
Age (years)	-	-	1.04 (0.93–1.16)	0.456	-	-
Female sex (%)	25 (89.3%)	65 (80.2%)	0.48 (0.31–1.81)	0.277	-	-
BMI (kg/m^2^)	-	-	0.89 (0.76–1.05)	0.202	-	-
Foot size	-	-	0.89 (0.73–1.09)	0.270	-	-
Metatarsal F. (%)						
Index plus	10 (35.7%)	40 (49.4%)	1.39 (0.58–3.32)	0.211	-	-
Index plus minus	13 (46.4%)	31 (38.3%)	1.54 (0.47–4.98)	0.448	-	-
Index minus	5 (17.9%)	10 (12.3%)	0.56 (0.23–1.38)	0.466	-	-
Digital F. (%)						
Greek	3 (10.7%)	11 (13.6%)	0.76 (0.19–2.96)	0.696	-	-
Egyptian	20 (71.4%)	58 (71.6%)	0.99 (0.38–2.56)	0.986	-	-
Square	5 (17.9%)	12 (14.8%)	1.25 (0.39–3.92)	0.702	-	-

**Table 4:** Predicted factors to change from pronated to neutral posture.

	Neutralise *n* = 15	No Neutralise *n* = 94	Univariable Analysis		Multivariable Analysis	
			OR (IC 95%)	*p*-Value	OR (IC 95%)	*p*-Value
Foot orthoses	11 (73.3%)	41 (43.6%)	3.55 (1.05–11.97)	0.041	3.13 (0.91–10.84)	0.068
Age (years)	-	-	1.04 (0.90–1.20)	0.536	-	-
Female sex (%)	14 (93.3%)	76 (80.9%)	0.30 (0.37–2.44)	0.237	-	-
BMI (kg/m^2^)	-	-	0.94 (0.77–1.15)	0.602	-	-
Foot size	-	-	0.91 (0.71–1.17)	0.474	-	-
Metatarsal F. (%)						
Index Plus	8 (53.3%)	36 (38.3%)	1.84 (0.61–5.51)	0.270	-	-
Index Plus Minus	4 (26.7%)	11 (11.7%)	2.74 (0.74–10.12)	0.118	-	-
Index Minus	3 (20%)	47 (50.0%)	0.25 (0.06–0.94)	0.048	0.28 (0.07–1.08)	0.065
Digital F. (%)						
Greek	1 (6.7%)	13 (13.8%)	0.44 (0.05–3.67)	0.687	-	-
Egypcian	13 (86.7%)	65 (69.1%)	2.90 (0.61–13.68)	0.163	-	-
Square	1 (6.7%)	16 (17.0%)	0.34 (0.4–2.84)	0.458	-	-

**Table 5:** FPI changes according to the metatarsal formula status in both groups.

		Index Plus (*n* = 44)		Index Minus Plus (*n* = 15)		Index Minus (*n* = 40)	
	Group	∆ FPI Score ± SD	*p*-Value	∆ FPI Score ± SD	*p*-Value	∆ FPI Score ± SD	*p*-Value
Talar head palpation	Control	0.25 ± 0.44	0.094	−0.28 ± 0.48	0.887	0.20 ± 0.48	0.754
	Experimental	0.00 ± 0.51		−0.25 ± 0.46		0.25 ± 0.63	
Supra and infra lateral malleolar curvature	Control	0.15 ± 0.58	0.370	0.00 ± 0.57	0.616	0.30 ± 0.53	0.081
	Experimental	0.00 ± 0.51		−0.12 ± 0.35		0.00 ± 0.64	
Calcaneal frontal plane position	Control	0.05 ± 0.60	0.022	0.14 ± 0.37	0.048	0.36 ± 0.71	0.198
	Experimental	−0.37 ± 0.57		−0.37 ± 0.51		0.05 ± 0.99	
Prominence in the region of the talonavicular joint	Control	0.20 ± 0.52	0.007	−0.14 ± 0.37	0.926	0.20 ± 0.61	0.161
	Experimental	−0.25 ± 0.53		−0.12 ± 0.35		−0.05 ± 0.60	
Congruence of the medial longitudinal arch	Control	0.20 ± 0.61	0.013	0.14 ± 0.37	0.048	0.20 ± 0.55	0.247
	Experimental	−0.25 ± 0.53		−0.37 ± 0.51		0.00 ± 0.64	
Abduction/adduction of the forefoot on the rearfoot	Control	0.20 ± 0.52	0.001	0.14 ± 0.69	0.111	0.26 ± 0.58	<0.001
	Experimental	−0.41 ± 0.65		−0.50 ± 0.75		−0.45 ± 0.60	
Total FPI score	Control	1.25 ± 1.83	<0.001	0.00 ± 1.29	0.037	1.53 ± 2.41	0.018
	Experimental	−1.45 ± 2.10		−1.75 ± 1.58		−0.20 ± 2.50	

## Data Availability

The dataset is available on request from the authors.
